# Knee movements cause changes in the firing behaviour of muscle spindles located within the mono‐articular ankle extensor soleus in the rat

**DOI:** 10.1113/EP090764

**Published:** 2023-02-24

**Authors:** Huub Maas, Wendy Noort

**Affiliations:** ^1^ Department of Human Movement Sciences, Faculty of Behavioural and Movement Sciences Vrije Universiteit Amsterdam Amsterdam The Netherlands; ^2^ Amsterdam Movement Sciences Amsterdam The Netherlands

**Keywords:** myofascial force transmission, primary afferent, proprioception, rat

## Abstract

We recently showed that within an intact muscle compartment, changing the length of one muscle affects the firing behaviour of muscle spindles located within a neighbouring muscle. The conditions tested, however, involved muscle lengths and relative positions that were beyond physiological ranges. The aim of the present study was to investigate the effects of simulated knee movements on the firing behaviour of muscle spindles located within rat soleus (SO) muscle. Firing from single muscle spindle afferents in SO was measured intra‐axonally for different lengths (static) and during lengthening (dynamic) of the lateral gastrocnemius and plantaris muscles. Also, the location of the spindle within the muscle was assessed. Changing the length of synergistic ankle plantar flexors (simulating different static knee positions, between 45 and 130°) affected the force threshold, but not the length threshold, of SO muscle spindles. The effects on type II afferents were substantially (four times) higher than those on type IA afferents. Triangular stretch–shortening of synergistic muscles (simulating dynamic knee joint rotations of 15°) caused sudden changes in the firing rate of SO type IA and II afferents. Lengthening decreased and shortening increased the firing rate, independent of spindle location. This supports our prediction that the major point of application of forces exerted by connections between adjacent muscles is at the distal end of SO. We conclude that muscle spindles provide the CNS with information about the condition of adjacent joints that the muscle does not span.

## Abstract (highlights)

- **What is the central question of this study?**
  Do length changes of bi‐articular neighbouring muscles affect the firing behaviour of a mono‐articular ankle plantar flexor?
- **What is the main finding and its importance?**
  Simulating knee movements by changing the length of synergistic ankle plantar flexors caused sudden changes in the firing rate of type IA and II afferents. This implies that muscle spindles provide the CNS with information about the condition of adjacent joints that the muscle does not span.

## 1 INTRODUCTION

It has been argued that muscle spindles play the key role in kinesthesia, defined as the sense of movement and of position of the body and its parts (Proske & Gandevia, 2012). When a limb moves or changes position, joint rotations result in changes in the length of several muscles and, thereby, affect the length of muscle spindles. Others have indicated that a combination of feedback from muscle spindles and Golgi tendon organs is required for accurate coding of muscle–tendon unit (MTU) length (Dimitriou & Edin, 2008; Kistemaker et al., 2013). That would be applicable in particular during conditions in which changes in muscle fascicle length differ from changes in MTU length (Griffiths, 1991; Maas & Lichtwark, 2009; Maas et al., 2009). In both cases, muscle receptors are assumed to provide information exclusively about the joint(s) spanned by the muscle in which they are located. This assumption was recently challenged by our observations that changing the length of a synergistic muscle affected the firing behaviour of muscle spindles and tendon organs located within a neighbouring muscle (Maas et al., 2022; Smilde et al., 2016). More specifically, lengthening a muscle spanning both the ankle and knee joints affected firing of receptors in a muscle spanning only the ankle joint. This suggests that feedback from muscle receptors located in a one‐joint muscle might contain information about the position of joints that the muscle does not span.

More than a century ago, Sherrington indicated with regard to the ‘receptor organs in the deep tissues’ that ‘the stimuli occurring in this deep field are traceable to actions of the organisms itself’, hence they might be termed ‘proprio‐ceptors’ (Sherrington, 1906). This led to the question regarding the nature of the mechanical stimulus to which muscle receptors respond (Sherrington, 1909). Despite more than a century of research on this topic (e.g., Banks, 2023; Matthews, 1972), there is still debate about the precise make‐up of the output of muscle spindles. ‘What do muscle spindles sense?’ has been identified as one of the principal unanswered questions in motor control (Nordin et al., 2017). Spindle firing rate was found to be correlated much more strongly with force and yank than with length and velocity in a passive muscle (Blum et al., 2017, 2019), disputing the classical concept of muscle spindles encoding length and velocity. In addition, it has been proposed that muscle spindles do not merely function as proprioceptors, but mediated by gamma motor control (i.e., spindle tuning), play an active role in sensorimotor performance (for details see Dimitriou, 2022).

A mechanism that might explain our recent findings (Maas et al., 2022; Smilde et al., 2016) is force transmission between muscles via linking connective tissues, so‐called epimuscular force transmission (Huijing, 2009; Maas & Sandercock, 2010). Forces exerted on a muscle by epimuscular linkages can result in local changes in length (Tijs et al., 2015a) and force (Maas, 2019). A condition facilitating this mechanism is one in which the position of a muscle is changed relative to an adjacent muscle for which the MTU length is kept constant (Maas et al., 2004). During normal movements, such a condition is found when the joint that is spanned by a bi‐articular muscle, but not by the adjacent mono‐articular muscle, is rotated. In our previous studies (Maas et al., 2022; Smilde et al., 2016), the effects of intermuscular interactions on the firing behaviour of muscle receptors were tested for rather uncommon, often unphysiological muscle lengths and relative positions. In addition, the effects of epimuscular linkages were assessed in a static way. This means that first the position of a synergistic muscle was altered before the ramp stretches to assess firing responses were imposed. Especially IA afferents are expected to be affected more during dynamic epimuscular loading. This might explain why, in our previous studies, the length and force threshold (i.e., the force and MTU length change at which the first action potential occurred), but not the peak firing rate and the firing rate during the hold phase, were altered.

The aim of the present exploratory study was to investigate the effects of simulated knee movements on the firing behaviour of muscle spindles located within rat soleus (SO) muscle, a mono‐articular ankle extensor. For this purpose, firing from single muscle spindle afferents in SO were measured intra‐axonally for different lengths (static) and during lengthening (dynamic) of the lateral gastrocnemius (LG) and plantaris (PL) muscles, ankle extensors and knee flexors (i.e., bi‐articular). Also, the location of the spindle within the muscle (proximal, middle or distal) was assessed. Static length changes were expected to affect predominantly type II afferents, whereas dynamic length changes were expected to change the firing rate in both type IA and II afferents.

**FIGURE 1:**
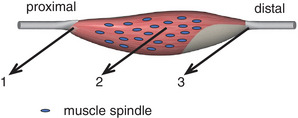
Schematic drawing of a muscle with spatially distributed muscles spindles (blue ovals). The three black arrows indicate the points of application of epimuscular forces corresponding to the three predictions (1, 2 and 3) on responses of muscles spindles depending on their location within the muscle (see main text).

The effects of intermuscular interactions on the firing behaviour will depend on the location of the muscle spindle within the muscle and on the location of the mechanical interactions between the muscles. This leads to the following predictions (Figure 1):

1. If the major point of application of epimuscular forces is at the proximal end of SO, lengthening of the neighbouring bi‐articular muscles (simulating knee extension) will result in an increase of the length and firing rate of all muscle spindles.
2. If the major point of application of epimuscular forces is in the middle of the SO muscle belly, lengthening of the neighbouring bi‐articular muscles will result in an increase of the length and firing rate of the more distally located muscle spindles, but in a decrease of the length and firing rate of the more proximally located spindles.
3. If the major point of application of epimuscular forces is at the distal end of SO, lengthening of the neighbouring bi‐articular muscles will result in a decrease of the length and firing rate of all muscle spindles.

## 2 METHODS

### 2.1 Ethical approval

Surgical and experimental procedures were in agreement with the guidelines and regulations concerning animal welfare and experimentation set forth by Dutch law and were approved by the Committee on Ethics of Animal Experimentation at the Vrije Universiteit Amsterdam (permit number: FBW 12‐02). The study was performed and reported according to the ‘principles and standards for reporting animal experiments’ (Grundy, 2015).

### 2.2 Overview of experimental design

In this study, the length and relative position of the two‐joint LG and PL muscles was manipulated proximally, simulating changes in knee joint angle, and the firing behaviour of muscle spindles in the one‐joint SO was assessed. First, effects of different simulated knee joint angles were tested statically. Second, effects of dynamic simulated knee movements were assessed.

### 2.3 Animals

Data were collected from 11 female Wistar rats (*Rattus norvegicus*, body mass = 254 ± 11 g), from the own breed of the animal facility of the Vrije Universiteit Amsterdam. All animals were housed in pairs with access to food and water ad libitum. Rats were anaesthetized by an i.p. injection of urethane (Sigma‐Aldrich) (initial dose: 1.2 ml/100 g body mass, 12.5% urethane solution; additional doses to maintain suppression of withdrawal reflexes: 0.2 ml). Animal core body temperature was maintained with heating pads and lamps. Subcutaneous injections of lactated Ringer solution (1 ml/h) were given to maintain fluid balance. At the end of the experiment, rats were killed with an overdose of intracardially injected pentobarbitone sodium (Euthasol 20%, Ast Farma B.V) followed by a double‐sided pneumothorax.

### 2.4 Surgical procedures

The surgery was equal to that of Experiment 2 described in a previous study (Maas et al., 2022). In brief, the lateral and medial gastrocnemius (MG), SO and PL muscles as a group were dissected free from their surroundings. The biceps femoris muscle and crural fascia were resected, but care was taken to ensure that the connective tissues between the muscle bellies were left intact. With the knee and ankle angles at 90°, sutures were placed on the proximal and distal tendons of LG, on the proximal tendon of the extensor digitorum longus muscle for the proximal reference position, and on the peroneus longus distal tendon for the distal reference position, defining the reference lengths and relative positions for SO and LG + PL (*L*_ref_). The MG subtendon (Finni et al., 2018) and muscle belly were dissected free. The distal tendons of LG, PL and SO were tied together close to the calcaneal bone (referred to as LG + PL + SO). The calcaneal bone was cut with LG and SO attached to it, and the PL tendon was cut distal to the calcaneal bone. Also, the proximal tendons of LG and PL were tied and cut (referred to as LG + PL).

The common peroneal, sural, plantar and MG nerves were isolated and crushed. A bipolar stimulating electrode was placed on the nerve branches of LG and SO. To prevent tissue dehydration, the muscles were irrigated regularly with saline.

To expose the dorsal roots, a back surgery was performed. First, a local anaesthetic (bupivacaine; B. Braun. 1 ml, 0.025%) was injected s.c. An incision from sacral vertebra S2 to thoracic vertebra Th10 was made in the skin. The spinous and articular processes were cleared of tissues in order that the spine could be clamped securely. A bath of mineral oil was constructed using skin flaps. A laminectomy was performed, and the dura mater was removed to expose the dorsal roots (L1–L5).

### 2.5 Experimental protocols

**FIGURE 2:**
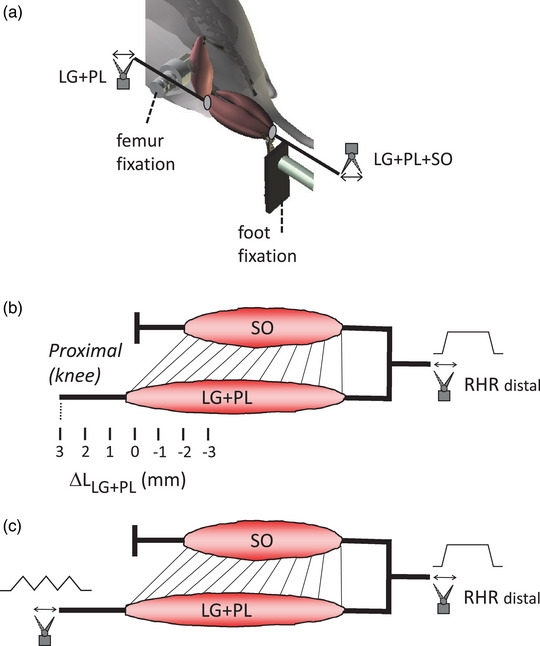
Overview of experimental set‐up and conditions. (a) Schematic drawing of the experimental set‐up. The distal tendons of the lateral gastrocnemius (LG), plantaris (PL) and soleus (SO) muscles as a group were attached to one servomotor (LG + PL + SO). The proximal tendons of LG and PL were attached to a second servomotor (LG + PL). (b) Diagram for the experiment on simulating static knee positions. Different lengths and positions of LG + PL relative to SO were imposed; Δ*L*_LG + PL_ = 0 refers to the reference length and position, corresponding to a knee and ankle angle of 90°. Connective tissue linkages between the muscles are illustrated schematically as straight lines. (c) Diagram for the experiment simulating dynamic knee movements. During a ramp–hold–release (RHR) imposed at the LG + PL + SO complex distally, triangular stretch–shortening cycles were imposed at LG + PL proximally. For further details, see main text.

The hindlimb was secured to the set‐up with knee and ankle angles of 90° (Figure 2a). The distal tendons of LG + PL + SO and the proximal tendons of LG + PL were attached to separate servomotors (Dual‐Mode Lever 309C; Aurora Scientific). During all measurements, the muscles were in a passive state.

For the experiment on simulating static knee positions, stretches in the form of a ramp–hold–release (RHR; velocity ramp and release 20 mm/s, amplitude 3 mm, hold phase 1 s) were applied to the ankle plantar‐flexion muscles distally. Lengthening LG + PL + SO by 3 mm corresponds approximately to a change in ankle angle of 45° (Bernabei et al., 2015). Three consecutive RHRs were applied, with 4 s rest in between. Ramps were applied at the shortest MTU length for which firing during the stretch and hold phase was found (maximally *L*_ref_ + 3 mm), yielding a mean starting length of *L*_ref_ + 2.4 ± 0.8 mm for type IA afferents and *L*_ref_ + 2.4 ± 0.8 mm for type II afferents. The RHRs of LG + PL + SO distally were applied at seven different lengths of LG + PL, as obtained by displacements of the proximal LG + PL tendons from *L*_ref_ − 3 mm to *L*_ref_ + 3 mm in increments of 1 mm (Figure 2b).

For the experiment simulating dynamic knee movements, a combination of a RHR stretch distally and triangular stretch–shortening proximally was applied. First, the LG + PL + SO complex was stretched distally (20 mm/s ramp, amplitude 3 mm, hold phase 4 s). After 0.5 s, three triangular stretch–shortening cycles were applied on LG + PL proximally (2 mm/s, amplitude 1 mm), followed by shortening of the LG + PL + SO (Figure 2c). Lengthening LG + SO by 1 mm corresponds approximately to a change in knee angle of 15° (Bernabei et al., 2015). This protocol was repeated, with 4 s rest in between. For all recordings, the starting length was *L*_ref_ + 3 mm for LG + PL + SO distal and *L*_ref_ for LG + PL proximal.

### 2.6 Afferent recordings and analysis of firing behaviour

All procedures were similar to those described previously (Maas et al., 2022; Smilde et al., 2016). In brief, action potentials were recorded intra‐axonally in the dorsal root containing muscle spindle afferents from SO with a glass micropipette (20–50 MΩ, 2 M potassium acetate). Axons were classified as muscle spindle afferents if they displayed a pause in firing during the rising phase of a twitch contraction. Afferents were classified as group IA when they fired at a high frequency at the onset of a ramp stretch and fired at the same frequency (≤250 Hz) as length vibrations (40 μm amplitude). If neither of the above‐described properties was observed, afferents were classified as group II. To confirm that the spindles were located in SO and to assess the position within the muscle (proximal, middle or distal), the muscle surface was probed.

Intra‐axonal records were amplified (×100) and low‐pass filtered (second order, 2–10 kHz; Axoprobe 1A; Axon Instruments, Sunnyvale, CA, USA). All signals were digitized (20 kHz; Power 1401; CED, Cambridge, UK) and stored on a computer for later analysis using Spike2 software (CED).

For the experiments on static knee positions, data from seven type IA and seven type II SO afferents were recorded. Changes in afferent firing behaviour were assessed by the force and length thresholds (i.e., change in force and MTU length at which the first action potential occurred upon stretch). Previously, these parameters were found to be most sensitive to changes in muscle relative position (Smilde et al., 2016). For comparison with the literature, also the maximum instantaneous firing rate (IFR) at the end of the dynamic stretch [peak frequency (PF)] and the IFR halfway through the hold phase [static index (SI)] were assessed. For data analysis, only the final two of the three repetitions were used to calculate means.

**FIGURE 3:**
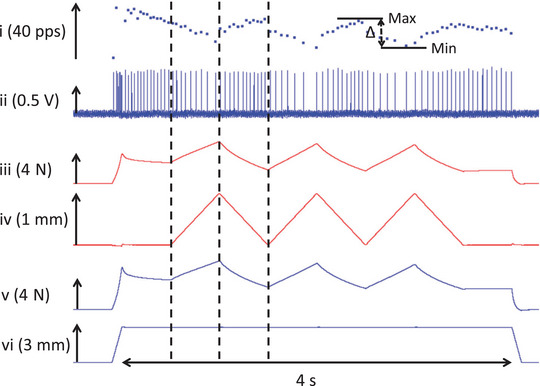
Exemplar recordings for the conditions in the experiment on dynamic knee movements. The signals, from top to bottom, are as follows: (i) instantaneous firing rate; (ii) action potentials; (iii) forces exerted at the LG + PL proximally; (iv) changes in length imposed at LG + PL proximally; (v) forces exerted at the LG + PL + SO distally; and (vi) changes in length imposed at LG + PL + SO distally. Vertical arrows indicate the *y*‐axis scale. Vertical dashed lines mark the lengthening and shortening phases of the first triangular stretch–shortening cycle of LG + PL proximally. Abbreviations: LG, lateral gastrocnemius; PL, plantaris; SO, soleus.

For the experiment on dynamic knee movements, afferent responses were recorded from nine type IA and eight type II SO afferents. The changes in firing rate (ΔIFR) during the stretch and the shortening phases of the three triangular cycles were assessed (Figure 3). The ΔIFR was calculated as the IFR at the start of stretch/shortening minus the IFR at the end of stretch/shortening. In some cases, afferent firing ceased. If there were no action potentials within the stretch or shortening phase, a ΔIFR was not calculated. For data analysis, only the final two triangles of each of the three repetitions were used to calculate means.

### 2.7 Statistics

Data were analysed using SPSS v.26 (SPSS, Chicago, IL, USA). For the experiment simulating static knee positions, repeated‐measures ANOVAs were performed (factor: length LG + PL proximally). If the assumption of sphericity was violated, assessed using Mauchly's sphericity test, a Greenhous–Geisser correction was applied. For the experiment simulating dynamic knee movements, Student's unpaired *t*‐tests were performed to assess whether the ΔIFR was different between type IA and II afferents. The statistically significant level was set at *P* = 0.05.

## 3 RESULTS

### 3.1 Experiment simulating static knee positions

Axonal conductional delays and ramp parameters, measured at reference length and muscle relative position (Table 1), were similar to those in our previous studies (Maas et al., 2022; Smilde et al., 2016). The approximate location within the muscle belly could be identified for all except one of seven type IA afferents and two of seven type II afferents.

**TABLE 1:** Firing characteristics of muscle) spindles (type IA and II afferents) in soleus muscle and tendon forces measured before and during distal ramp‐hold‐releases of LG + PL + SO with the proximal tendon of LG + PL at *L*_ref_.

**Characteristic**	**Type IA afferents (*n* = 7)**	**Type II afferents (*n* = 7)**
Proximal–distal position	5 d, 1 m, 0 p, 1 nc	4 d, 1 m, 0 p, 2 nc
Axonal conductional delay (ms)	1.9 ± 0.3	2.1 ± 0.2
**Parameters at *L*_ref_**		
Force threshold (N)	0.35 ± 0.12	0.91 ± 0.55
Length threshold (mm)	0.10 ± 0.03	0.94 ± 0.91
Peak frequency (pps)	63 ± 20	56 ± 26
Static index (pps)	9 ± 10	13 ± 12
Pre‐ramp force distal (N)	0.26 ± 0.11	0.30 ± 0.15
Maximal ramp force distal (N)	3.51 ± 1.85	4.06 ± 2.51

Abbreviations: d, distal; LG, lateral gastrocnemius; m, middle; *n*, number of afferents; nc, not classified; p, proximal; pps, pulses per second; PL, plantaris; SO, soleus.

*Note*: Values are means ± SD.

**FIGURE 4:**
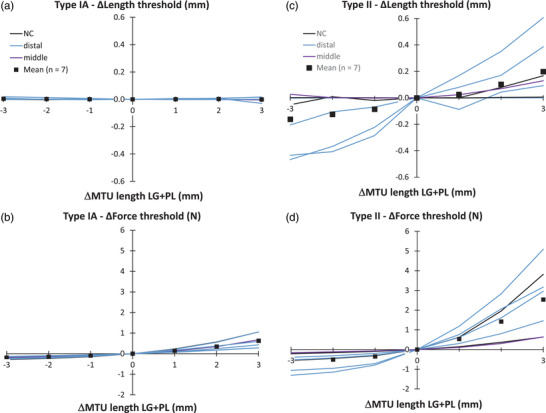
Effects of lengthening LG + PL proximally on length and force threshold of SO muscle spindles. (a) Length threshold of type IA afferents. (b) Force threshold of type IA afferents. (c) Length threshold of type II afferents. (d) Force threshold of type II afferents. Each parameter is plotted as a function of the MTU length of LG + PL, expressed as the deviation from *L*_ref_ (ΔMTU length). To compare individual cells, the value at *L*_ref_ (see Table 1) was subtracted from the value at all other lengths to obtain a delta (Δ) value. Mean values (*n* = 7 for both types) are shown as black squares. Different colours indicate the position of the muscle spindle within the muscle belly (keys in a and c). Abbreviations: LG, lateral gastrocnemius; *L*_ref_, reference length; MTU, muscle–tendon unit; PL, plantaris; SO, soleus.

The length threshold of type IA afferents of SO was not affected by LG + PL length (*P* = 0.581), but a significant (*P* = 0.001) increase (by 0.82 N) in the force threshold was found (Figure 4). Changing LG + PL length affected type II afferents substantially more (by 3.8 N) than type IA afferents (Figure 4). The effects on the force threshold were significant (*P* = 0.006), whereas those on the length threshold were not (*P* = 0.056). Note the large variability between afferents.

### 3.2 Experiment simulating dynamic knee movements

The approximate location within the muscle belly could be identified for all except one of nine type IA afferents and four of eight type II afferents. Six of the localized type IA afferents appeared to be positioned distally, and two appeared to be positioned in the middle of the muscle belly. All four localized type II afferents appeared to be positioned distally.

**FIGURE 5:**
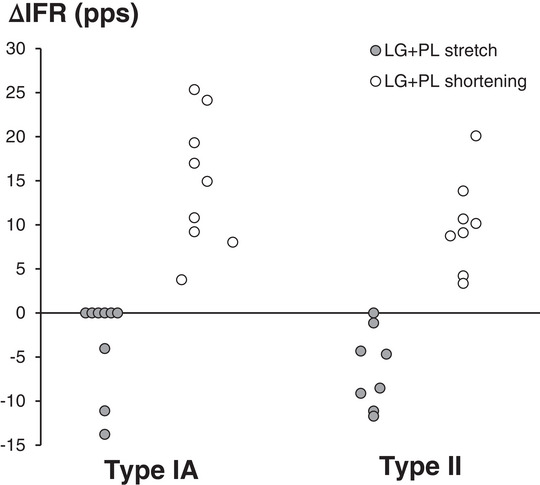
Changes in instantaneous firing rate (ΔIFR) of SO muscle spindles in response to triangular stretch–shortening of LG + PL proximally. Individual data for type IA (*n* = 9) and type II (*n* = 8) afferents are shown. The value of ΔIFR was calculated as the IFR at the start of stretch/shortening minus the IFR at the end of stretch/shortening. During stretch, IFR decreased (hence the negative values for ΔIFR) or firing paused (circles on *x*‐axis, ΔIFR = 0). During shortening, IFR increased (hence the positive values for ΔIFR). In cases when the afferent ceased firing and there were no action potentials within the stretch or shortening phase, no ΔIFR was calculated. In the graph, these cases are pictured as circles with a value of zero. Abbreviations: LG, lateral gastrocnemius; PL, plantaris; SO, soleus.

**FIGURE 6:**
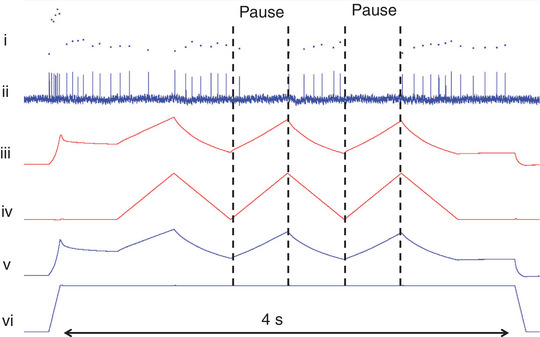
Exemplar recording of a muscle spindle that paused firing in response to LG + PL lengthening. The signals from top to bottom are as follows: (i) instantaneous firing rate; (ii) action potentials; (iii) forces exerted at the LG + PL proximally; (iv) changes in length imposed at LG + PL proximally; (v) forces exerted at the LG + PL + SO distally; and (vi) changes in length imposed at LG + PL + SO distally. Abbreviations: LG, lateral gastrocnemius; PL, plantaris; SO, soleus.

All muscle spindles within SO reacted to the triangular stretch–shortening of LG + PL proximally (Figure 5). For type IA afferents, LG + PL stretch resulted in a decrease (i.e., a negative ΔIFR, between −4 and −14 pps; pulses per second) in the IFR (Figures 3 and 5) or pausing of firing (indicated as zero change in Figure 5; also see Figure 6). Shortening caused an increase (i.e., a positive ΔIFR, between 4 and 25 pps) of the IFR. For type II, only one afferent paused during LG + PL lengthening. All others decreased firing (between −1 and −12 pps). Similar to type IA, type II afferents increased firing in response to LG + PL shortening (between 3 and 20 pps). Effects of stretch (*P* = 0.433) and shortening (*P* = 0.158) were not different between type IA and II afferents.

## 4 DISCUSSION

This is the first study in which effects of connective tissue linkages between mono‐articular soleus and the bi‐articular synergistic muscles on muscle spindle firing behaviour have been assessed for dynamic and physiological muscle conditions (i.e., those simulating knee movements). Our main findings are as follows: (1) changing the length of synergistic ankle plantar flexors affected the force threshold of muscle spindles of SO; as hypothesized, the effects on type II afferents were substantially larger than those on type IA afferents; and (2) triangular stretches simulating knee joint rotations caused sudden changes in the firing rate of type IA and II afferents.

In our previous studies assessing effects of intermuscular linkages on firing behaviour of muscle spindles and tendon organs, we found effects on the force and length thresholds but not on the firing rate (Maas et al., 2022; Smilde et al., 2016). The main difference from previous studies is that in the present study, effects of dynamic length changes (i.e., triangular stretch–shortening cycles) of neighbouring muscles were assessed. Given that the structures involved in these epimuscular effects consist of connective tissue, their properties can be described as viscoelastic. When changing the position of the synergistic muscle is performed before the ramp stretch of the agonist muscle, the forces of the intermuscular linkages are probably already reduced owing to effects of stress relaxation. When these linkages are strained at a certain velocity, the viscous properties result in increased forces. This indicates that effects of epimuscular linkages are enhanced during dynamic loading, thus explaining that effects on spindle firing rate were observed. There is only one previous report regarding effects of epimuscular force transmission during dynamic changes in muscle length (Maas & Huijing, 2005), but direct comparisons between static and dynamic muscle conditions have not yet been made.

We hypothesized that the epimuscular effects on muscle spindle firing behaviour would depend on the location of the spindle within the muscle and on the location of the net point of application of epimuscular forces (Figure 1). Most muscle spindles measured in the present study were located distally and a few in the middle of SO. Given that the responses to lengthening of the neighbouring bi‐articular muscles (simulating knee extension) were in the same direction for all spindles (i.e., decrease in firing rate or pausing of firing), our results support prediction 3 that the major point of application of epimuscular forces is at the distal end of SO. This agrees with previous observations that the stiffness of connective tissues between SO and gastrocnemius varies along the proximodistal axis, being the highest distally (Maas & Sandercock, 2010). It should be noted that similar effects could be brought about by mechanical interactions via a shared in‐series elasticity (i.e., the Achilles tendon). Although some independent movement of the subtendons within the proximal region of the rat Achilles tendon is possible (Finni et al., 2018; Maas et al., 2020), we also found that knee extension with all muscles in a passive state increased the length of the distal region of the Achilles tendon (Tijs et al., 2015b). With a constant MTU length of SO, lengthening of the distal tendon will result in shortening of its muscle belly. In agreement, knee extension in humans caused shortening of SO muscle fascicles in combination with lengthening of the Achilles tendon (Tian et al., 2012). Thus, our results are likely to be mediated by interactions via both intermuscular connective tissues and the shared portion of the Achilles tendon.

In the present study, only passive muscle conditions were tested. Selective activation of the bi‐articular muscles will not only result in more lengthening of the Achilles tendon (Tijs et al., 2014), but also in increased epimuscular force transmission (Tijs et al., 2016). At the same time, changes in γ‐motoneuron drive will affect the firing responses of muscle spindles (Matthews, 1964). As γ‐motoneuron drive is actively modulated during normal behaviour (Dimitriou, 2022), the effects of intermuscular connective linkages on spindle firing observed in our study might differ from those in the freely moving animal. As discussed previously (Smilde et al., 2016), γ‐motoneuron control might correct (override) or enhance signals related to epimuscular forces. If this is the case, is currently unknown. However, we did find changes in the recruitment strategy in response to an increase in the stiffness of connective tissue between SO and LG (Bernabei et al., 2017), suggesting that the CNS takes effects of intermuscular interactions into account.

Muscle spindles are spatially distributed within the muscle belly (Arendt & Asmussen, 1974; Swett & Eldred, 1960). ‘Why do skeletal muscles have so many and so dispersed muscle spindles?’ is an unsolved question (Banks, 2015; Windhorst, 2008). One of the recently proposed (Maas et al., 2022) answers is that this allows them to provide feedback about strains in a specific location. Epimuscular forces might cause non‐uniform strains within a muscle (Tijs et al., 2015a). The large variability in responses of SO muscle spindles to changes in the length of neighbouring muscles observed in the present study suggests such sensing of local muscle conditions. With the relatively small sample size and the limited distribution of tested afferents, we have to be cautious with drawing strong conclusions about this. However, our results do provide strong evidence that muscle spindles sense changes in length of neighbouring muscles. This allows us to conclude that muscle spindles might provide the CNS with information about the condition of adjacent joints that the muscle does not span.

In addition to the integration of single‐joint sensory feedback (Bosco et al., 2000; Pruszynski et al., 2011), such intrinsic multi‐joint information might help the CNS with whole‐limb neuromuscular control. This hypothesis agrees with the results of a study in which knee rotation affected not only the H reflex amplitude of the bi‐articular MG, but also that of the mono‐articular SO (Tokuno et al., 2012), and with the finding that the gain of stretch reflexes of muscles acting on the shoulder was affected by the level of background activity of other surrounding muscles (Nicolozakes et al., 2022). In both studies, the results might also be explained by neural coupling between muscles (Nichols, 2018). However, with the experimental methods applied, the role of mechanical and neural coupling cannot be distinguished. Further studies are needed to assess effects intermuscular mechanical interactions on sensory feedback in the intact animal (i.e., in the presence of γ‐motoneuron control) and to study the potential impact in the neural control of movement.

## AUTHOR CONTRIBUTIONS

Conception or design of the work: Huub Maas. Acquisition, analysis, or interpretation of data for the work: Huub Maas and Wendy Noort; Drafting of the work or revising it critically for important intellectual content: Huub Maas and Wendy Noort. Both authors approved the final version of the manuscript and agree to be accountable for all aspects of the work in ensuring that questions related to the accuracy or integrity of any part of the work are appropriately investigated and resolved. Both persons designated as authors qualify for authorship, and both those who qualify for authorship are listed.

## CONFLICT OF INTEREST

None declared.

## Supporting information

Statistical Summary Document

## Data Availability

The data that support the findings of this study are available from the corresponding author upon reasonable request.
